# Clinical features, risk factors, and clinical burden of acute kidney injury in older adults

**DOI:** 10.1080/0886022X.2020.1843491

**Published:** 2020-11-16

**Authors:** Yanhua Wu, Wenke Hao, Yuanhan Chen, Shaohua Chen, Wei Liu, Feng Yu, Wenxue Hu, Xinling Liang

**Affiliations:** Division of Nephrology, Guangdong Provincial People’s Hospital, Guangdong Academy of Medical Sciences, Guangdong Geriatrics Institute, Guangzhou, China

**Keywords:** acute kidney injury, geriatric, dementia, tumor, epidemiological study

## Abstract

***Background:*** Few epidemiologic studies on acute kidney injury (AKI) have focused on the older adult population. This study investigated the clinical features, risk factors, and clinical burden in this population.

***Methods:*** A retrospective observational study was performed with the clinical data of inpatients at Guangdong Geriatrics Institute from 1 August 2012, to 31 December 2016. AKI was classified into community-acquired AKI (CA-AKI) and hospital-acquired AKI (HA-AKI), and the risk factors for AKI were ranked by weight. The relationships between AKI and adverse outcomes during hospitalization were analyzed using univariate and multivariate logistic regression.

***Results:*** In total, 6126 patients were enrolled, and 1704 patients developed AKI (27.8%): 6.3% had CA-AKI, and 21.5% had HA-AKI. In total, 1425 (23.3%), 202 (3.3%), and 77 (1.3%) patients had stage 1, 2 and 3 AKI, respectively. Age, dementia, moderate/severe renal disease, moderate/severe liver disease, metastatic solid tumor, female sex, congestive heart failure, chronic pulmonary disease, diabetes mellitus with chronic complications, non-metastatic tumor and lymphoma were independent risk factors for HA-AKI. The first five were also independent risk factors for CA-AKI. After multiple adjustment, AKI was associated with intensive care admission (CA-AKI: OR 5.688, 95% CI 3.122–10.361; HA-AKI: OR 4.704, 95% CI 3.023–7.298) and in-hospital mortality (CA-AKI: OR 5.073, 95% CI 2.447–10.517; HA-AKI: OR 13.198, 95% CI 8.133–21.419).

***Conclusion:*** AKI occurs in >25% of older adults in the geriatric ward. In addition to traditional risk factors, dementia and tumors were risk factors for AKI in older adults. AKI is closely related to a poor prognosis.

## Background

Aging of the population is a global issue, and in China, this problem has become increasingly prominent. By 2050, there will be 400 million Chinese citizens over 65 years old, 150 million of whom will be over 80 years old [1]. This situation would place a heavy burden on medical and health care.

Acute kidney injury (AKI) is a common clinical syndrome. Older patients have an increased risk of AKI due to a natural decline in renal function, increased comorbidities, and polypharmacy [2,3]. As a result, the risk of progression to chronic kidney disease (CKD), dialysis, and mortality after AKI is also increasing in this population [4,5]. According to a recent meta-analysis, AKI occurs in 21% of hospitalized patients worldwide [6]. The recent Global Snapshot study further described the prevalence of AKI in the world [7]. However, the epidemiological characteristics of AKI in the older adults are not well understood. AKI is classified into community-acquired (CA)-AKI or hospital-acquired (HA)-AKI according to where the patients are when they develop AKI. The etiology, clinical characteristics, and outcomes are substantially different between these two types of AKI [8,9]. However, AKI has not been stratified by category for analysis in the elderly population. In addition, the recognized risk factors in older adults, which include hypertension, diabetes, cardiovascular diseases, infection, and trauma, are similar to those in the general population [10]. Very few studies have addressed the relationship between AKI and diseases that are more common in older adults, such as dementia, hemiplegia, and tumors. Clarification of the specific risk factors and clinical hazards of AKI in older adults will help improve the comprehensive management of this disease. Furthermore, the effects of AKI on the clinical burden in older hospitalized patients is controversial. In older adults who underwent major surgery, a higher postoperative AKI stage was associated with increased in-hospital mortality only in patients ≤76 years but not in patients >76 years [11,12].

The Guangdong Geriatrics Institute is the largest center for the diagnosis and treatment of geriatric diseases in South China. Data from this institute was analyzed to help us understand the clinical features, risk factors, and clinical burden of AKI in older hospitalized adults.

## Materials and methods

### Study design and data collection

The cutoff age for the classification of older adults is 60 years old in China, according to the Chinese law of the Protection of the Rights and Interests of the Elderly (https://www.ilo.org/dyn/natlex/natlex4.detail?p_isn=71658&p_lang=en). In this retrospective observational study, the electronic health care data of 8,760 inpatients (over 60 years old) at Guangdong Geriatrics Institute were collected from August 1, 2012, to December 31, 2016; 7,670 patients (88%) had serum creatinine levels measured at least twice within 7 days. The exclusion criteria included stage 5 CKD or a baseline serum creatinine value > 353.6 μmol/L (4 mg/dL, based on the fact that preexisting CKD and AKI would be difficult to distinguish from each other in such patients), a maximum serum creatinine < 53 μmol/L (0.6 mg/dL) during hospitalization, previous amputation, or a hospital stay longer than 30 days [13]. Ultimately, 6,126 patients were included for analysis.

Baseline clinical and demographic data collected from all patients included age, sex, admission wards, comorbidities, whether dialysis was required, whether intensive care was required, hospital stay and in-hospital costs. In-hospital costs were converted according to the average China-USA exchange rate (1 CHY = 0.1471 dollars) in 2016.

The main comorbidity analysis was focused on common diseases in older adults. The Charlson comorbidity index is an assessment system for predicting the risk of death [14], and our previous studies have confirmed its value in older adult inpatients [15]. The comorbidities were classified based on the diagnosis records and then confirmed by trained nephrologists, as in our recent report [16]. Because of the importance of dementia in gerontology, our Geriatrics Institute has adopted a standard protocol for the diagnosis of dementia, which is determined by a neurologist based on the patient's clinical features, CT scan findings, electroencephalogram findings and activities of daily living score. If necessary, an examination of the cerebrospinal fluid is considered. Since the hospital does not admit or treat AIDS patients, the AIDS option in the Charlson comorbidity score was removed.

### Definitions of the investigated indices

AKI was determined and graded according to the definition in the 2012 KDIGO guidelines. AKI was defined as a serum creatinine level increased by 26.5 μmol/L (0.3 mg/dL) within 48 h or 50% within 7 days based on the baseline levels. Grade 1 AKI was defined as an increase in the serum creatinine level that was 1.5–1.9 times the baseline value; grade 2 was defined as an increase in the serum creatinine level that was 2–2.9 times the baseline; and grade 3 AKI was defined as an increase in the serum creatinine level that was more than 3 times the baseline or the need for renal replacement therapy [2]. CA-AKI was defined as serum creatinine levels at the time of admission that were 1.5 times higher than the minimum serum creatinine levels during hospitalization. Patients who developed AKI but did not meet community-acquired CA-AKI criteria were identified as having HA-AKI [17]. Patients who did not meet the above criteria or had fewer than two serum creatinine measurements performed in 7 days were included in the non-AKI group [13]. The estimated glomerular filtration rate (eGFR) was calculated according to the Chronic Kidney Disease Epidemiology Collaboration (CKD-EPI) equation [18].

### Statistical analysis

Statistical analysis was performed using SPSS 25.0 software. Normally distributed continuous measurement data are expressed as the mean ± standard deviation, and the comparison between groups was tested with a variance test. Nonnormally distributed continuous variables are expressed as the median [25th percentile, 75th percentile (Q25, Q75)], and the comparison between groups was tested with the rank-sum test. Count data are expressed as the number of cases (%), and the difference between groups was compared using the chi-square test. If at least one cell had an expected value less than 1, Fisher’s exact test was performed. The p values for multiple comparisons were adjusted by the Bonferroni method. Statistically significant variables in the univariate analysis (*p* < 0.05) were included in a multivariate logistic regression analysis to calculate the odds ratios (*ORs*) and 95% confidence intervals (95% CIs). A two-tailed *P*-value <0.05 was set as statistically significant.

## Results

### General information

**Figure 1.**
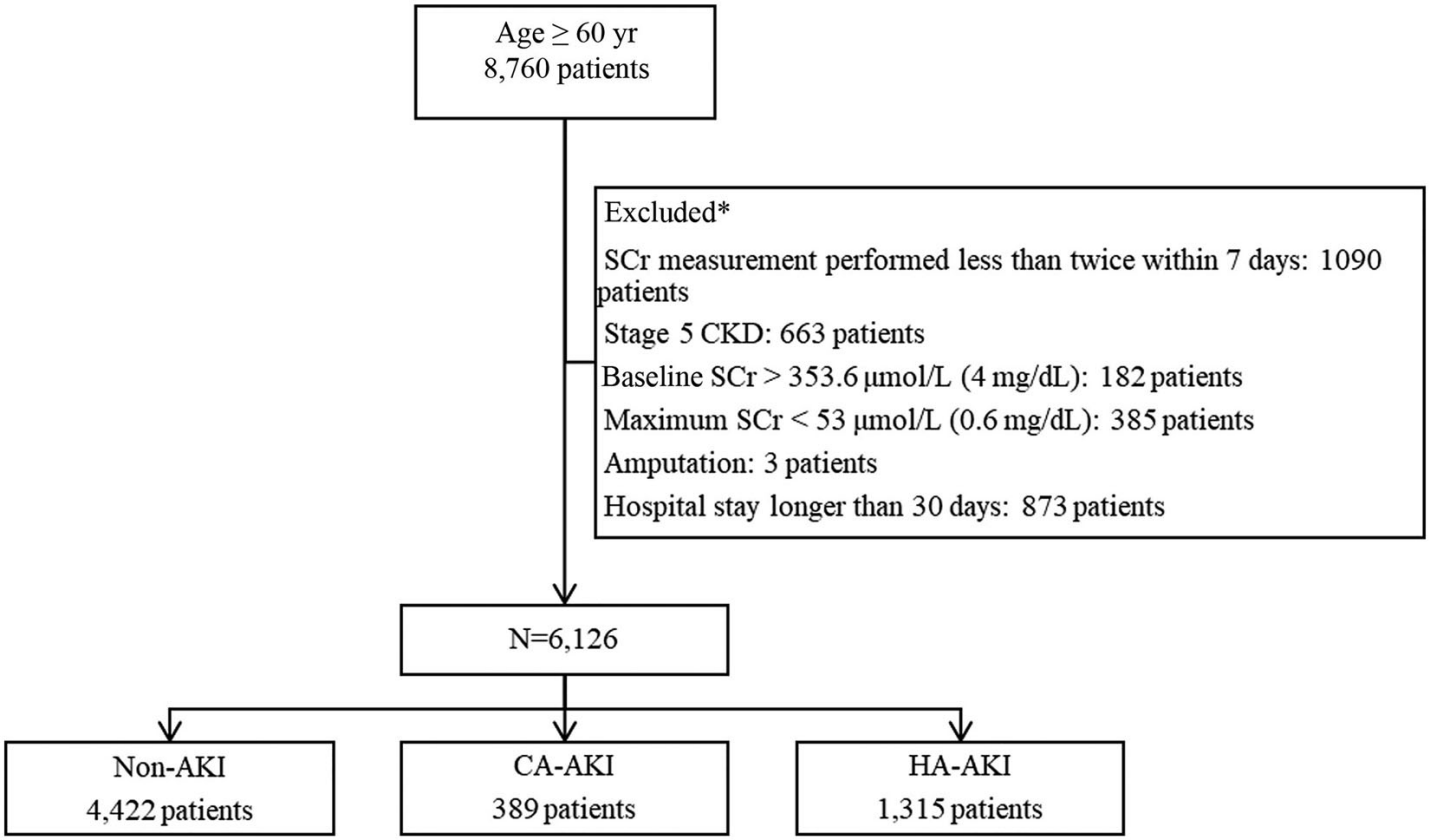
Flow chart of the selection of the study population. SCr: serum creatinine; CKD: chronic kidney injury; AKI: acute kidney injury; CA: community-acquired; HA: hospital-acquired; *Patients may met two or more exclusion criteria, such as patients with stage 5 chronic kidney disease who have been hospitalized for more than 30 days; therefore, a total of 2,634 patients were excluded.

A total of 1,704 (27.8%) patients with AKI were included in the study, including 1315 (21.5%) with HA-AKI and 389 (6.3%) with CA-AKI (Figure 1). AKI stages 1, 2 and 3 accounted for 1425 (23.3%), 202 (3.3%) and 77 (1.3%) cases, respectively. The incidence of AKI in the nephrology, respiratory medicine, gastroenterology, neurology, endocrinology and cardiology wards was 38.9%, 32.0%, 30.6%, 23.8%, 19.2% and 17.0%, respectively (Table 1). The ages, Charlson comorbidity index, and comorbidities in each ward are shown in Table S1.

**Table 1.** Major clinical characteristics of the study subjects.

	Non-AKI (*n* = 4,422)	CA-AKI (*n* = 389)	HA-AKI (*n* = 1,315)	*p* Value
Male, *N* (%)	3435 (77.7)	289 (74.3)	978 (74.4)^b^	0.022
Age [median (Q25, Q75)]	83 (77–87)	84 (81–88)^a^	85 (82–88)^b^	<0.001
60–74 years	871 (19.7)	40 (10.3) ^a^	119 (9.0) ^b^	<0.001
75–89 years	2962 (67.0)	282 (72.5)	940 (71.5)b	
≥90 years	589 (13.3)	67 (17.2)	256 (19.5)b	<0.001
**Ward, *N* (%)**				<0.001
Nephrology	440 (10.0)	61 (15.7)^a^	219 (16.7)^b^	
Respiratory medicine	1240 (28.0)	140 (36.0)^a^	444 (33.8)^b^	
Gastroenterology	894 (20.2)	85 (21.9)	310 (23.6)^b^	
Endocrinology	444 (10.0)	35 (9.0)	104 (7.9)	
Neurology	573 (13.0)	46 (11.8)	90 (6.8)^b^	
Cardiology	831 (18.8)	226 (5.7)^ac^	148 (11.3)^b^	
eGFR (ml/min/1.73 m^2^) [median (Q25, Q75)]	78 (61–86)	81 (59–91) ^a^	70 (44–85) ^b^	<0.001
Max Scr (µmol/L) [median (Q25, Q75)]	89 (73–110)	127 (92–189) ^ac^	116 (86–171) ^b^	<0.001
Min Scr (µmol/L) [median (Q25, Q75)]	76 (63–94)	67 (52–94) ^ac^	82 (62–119) ^b^	<0.001
Charlson comorbidity index [median (Q25, Q75)]	4 (2–5)	4 (3–6) ^a^	4 (3–7) ^b^	<0.001
**Comorbidities, *N* (%)**				
Hypertension	2955 (66.8)	268 (68.9)	950 (72.2)^b^	0.001
Congestive heart failure	798 (18.0)	78 (20.1)	315 (24.0)^b^	<0.001
Dementia	388 (8.8)	52 (13.4)^a^	212 (16.1)^b^	<0.001
Chronic pulmonary disease	1443 (32.6)	121 (31.1)	498 (37.9)^b^	0.001
Connective tissue disease	189 (4.3)	26 (6.7)	88 (6.7)^b^	<0.001
Moderate/severe renal disease	417 (9.4)	79 (20.3)^a^	260 (19.8)^b^	<0.001
Diabetes mellitus with chronic complications	175 (4.0)	23 (5.9)	73 (5.6)^b^	0.016
Non-metastatic tumor	806 (18.2)	87 (22.4)	400 (30.4)^b^	<0.001
Lymphoma	39 (0.9)	5 (1.3)	32 (2.4)^b^	<0.001
Moderate/severe liver disease	46 (1.0)	17 (4.4)^a^	60 (4.6)^b^	<0.001
Metastatic solid tumor	210 (4.7)	30 (7.7)^a^	131 (10.0)^b^	<0.001

AKI: acute kidney injury; CA: community-acquired; HA: hospital-acquired; eGFR: estimated glomerular filtration rate; SCr: serum creatinine;

^a^Significant difference between CA-AKI and non-AKI.

^b^Significant difference between HA-AKI and non-AKI.

^c^Significant difference between CA-AKI and HA-AKI.

The continuous variables, such as age, eGFR, Charlson comorbidity index score, max Scr and min Scr, were nonnormally distributed, and intergroup comparisons were performed with the rank-sum test, and the reported p values were adjusted by the Bonferroni method.

### Risk factors for AKI

**Figure 2.**
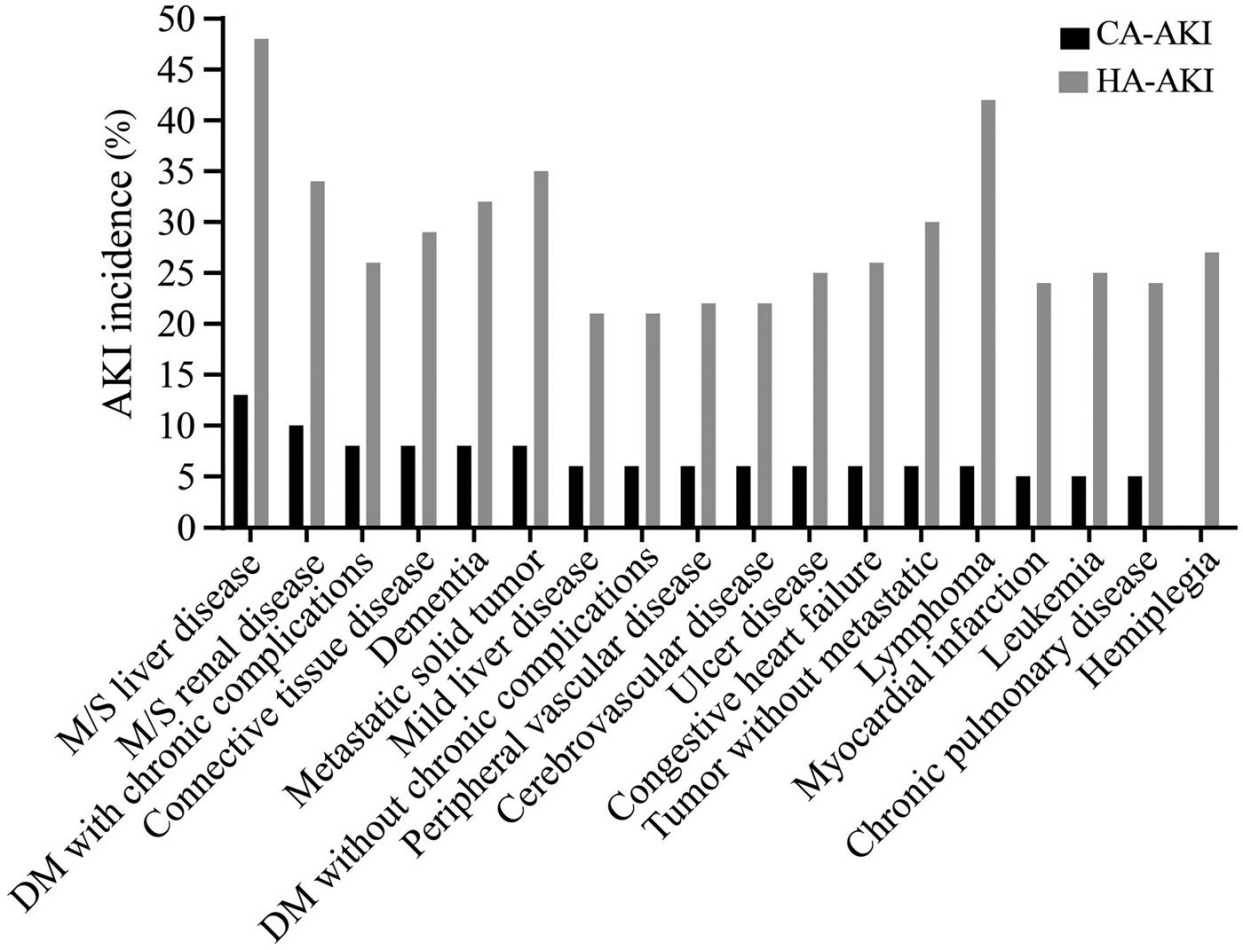
Incidence of AKI among patients with various clinical comorbidities. AKI: acute kidney injury; CA: community-acquired; HA: hospital-acquired.

The three comorbidities associated with the highest incidence of CA-AKI were moderate/severe liver disease (13.8%), moderate/severe renal disease (10.4%) and connective tissue disease (8.6%). For HA-AKI, the top three comorbidities were moderate/severe liver disease (48.8%), lymphoma (42.1%) and metastatic solid tumor (35.3%) (Figure 2).

**Figure 3.**
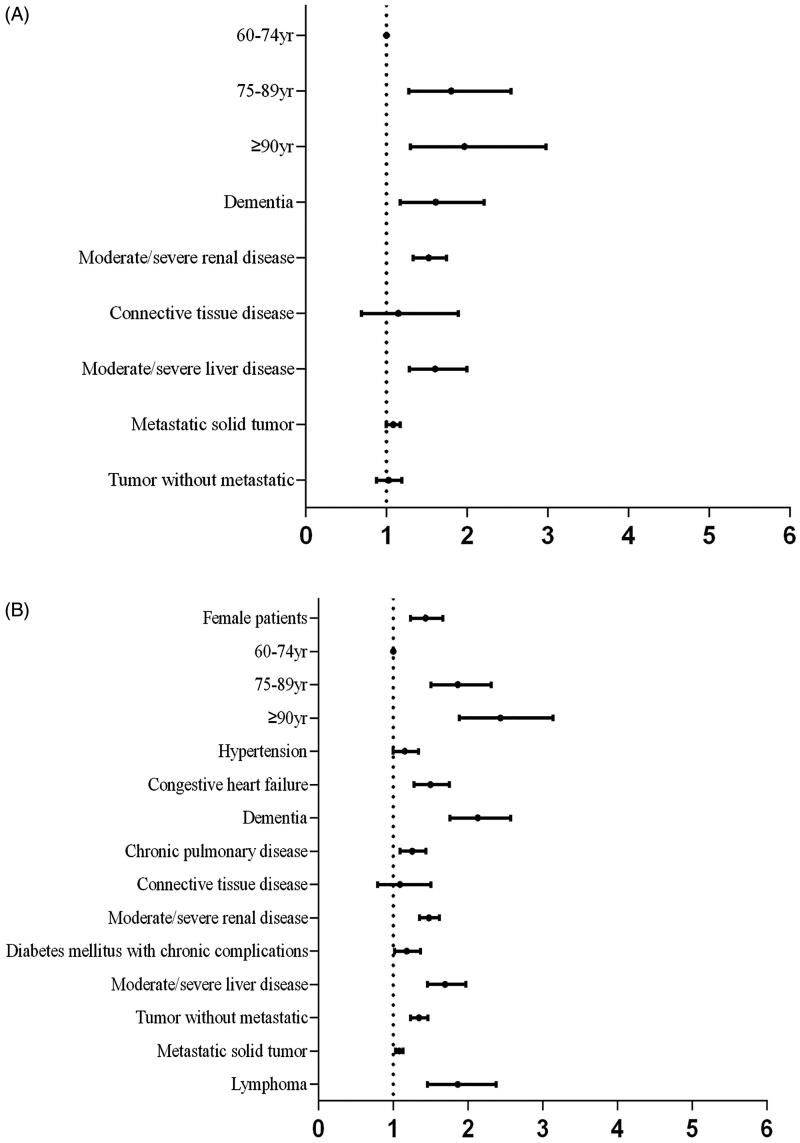
Relational analysis with multivariate logistic regression. A, Risk factors and CA-AKI. Variables that were different between patients with and without CA-AKI (*p* < 0.05) were selected for multivariate logistic regression to investigate the independent relationships between the related factors and CA-AKI. B, Risk factors and HA-AKI. Variables that were different between HA-AKI and non-AKI patients (*p* < 0.05) were selected for multivariate logistic regression to investigate the independent relationships between the related factors and HA-AKI.

Statistically significant variables in univariate analysis (Table 1) were included in a multivariate logistic regression analysis. Multivariate regression analysis showed that the independent risk factors for CA-AKI were age, dementia, moderate/severe renal disease, moderate/severe liver disease and metastatic solid tumor (Figure 3(A)) and that the independent risk factors for HA-AKI were female sex, advanced age, congestive heart failure, dementia, chronic pulmonary disease, moderate/severe renal disease, diabetes mellitus with chronic complications, non-metastatic tumor, lymphoma, moderate/severe liver disease and metastatic solid tumor (Figure 3(B)).

### AKI and in-hospital burden

**Figure 4.**
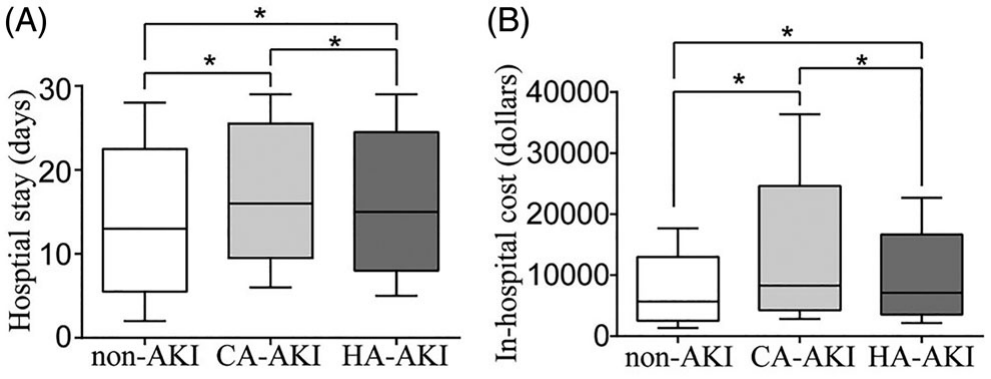
Relationships between AKI and hospital stay (A) and hospital cost (B). AKI: acute kidney injury; CA: community-acquired; HA: hospital-acquired; The horizontal bar denotes the median value, the box plot denotes the 25th and the 75th percentiles, and the whiskers denote the 2.5th and 97.5th percentiles.

For both the CA-AKI group and HA-AKI group, the length of hospital stay and the in-hospital cost were higher than those in the non-AKI group (Figure 4). The rates of requirement for dialysis, requirement for intensive care and in-hospital death in the AKI group were 1.7%, 5.0% and 8.5%, respectively, and were higher than those in the non-AKI group (*p* < 0.05).

After adjusting for factors such as the need for dialysis, myocardial infarction, cerebrovascular disease, dementia, mild liver disease, non-metastatic tumor, moderate/severe liver disease and metastatic solid tumor (supplementary Table S2), AKI increased the risk of the need for intensive care (CA-AKI: OR5.688, 95% CI 3.122–10.361; HA-AKI: OR 4.704, 95% CI 3.032–7.298) (Table 2).

**Table 2.** Risk factors for intensive care and in-hospital mortality in multivariate logistic regression models.

	Intensive care		P-value	In-hospital mortality		*p* Value
	OR	95% CI		OR	95% CI	
Female	–			–		
Age (years)						
60–74	–			Reference		0.365
75–89	–			1.530	0.806–2.905	0.194
≥ 90	–			1.670	0.798–3.515	0.173
AKI						
Non-AKI	Reference		<0.001	Reference		<0.001
CA-AKI	5.688	3.122–10.361	<0.001	5.073	2.447–10.517	<0.001
HA-AKI	4.704	3.032–7.298	<0.001	13.198	8.133–21.419	<0.001
Events						
Required dialysis	45.328	18.317–112.166	<0.001	34.187	10.906–107.162	<0.001
Required intensive care	–			15.007	8.871–25.385	<0.001
Comorbidities						
Hypertension	–					
Myocardial infarction	3.432	1.989–5.923	<0.001			
Congestive heart failure	–	–				
Peripheral vascular disease	–	–				
Cerebrovascular disease	0.473	0.315–0.712	<0.001			
Dementia	0.508	0.213–1.212	0.127			
Chronic pulmonary disease	–					
Connective tissue disease	–			0.747	0.305–1.831	0.524
Ulcer disease	–					
Mild liver disease	0.653	0.442–0.965	0.033			
Diabetes mellitus without chronic complications	–					
Hemiplegia	–					
Moderate/severe renal disease	–			1.011	0.797–1.284	0.926
Diabetes mellitus with chronic complications	–					
Non-metastatic tumor	1.163	0.907–1.492	0.233	1.143	0.889–1.471	0.297
Leukemia	–					
Lymphoma	–					
Moderate/severe liver disease	1.007	0.728–1.393	0.965	1.406	1.058–1.867	0.019
Metastatic solid tumor	1.091	0.976–1.220	0.126	1.437	1.314–1.572	<0.001

AKI: acute kidney injury; CA: community-acquired; HA: hospital-acquired; OR: odds ratio; 95% CI: confidence interval.

After adjusting for age, the need for dialysis, the need for intensive care, connective tissue disease, moderate/severe renal disease, non-metastatic tumor, moderate/severe liver disease and metastatic solid tumor (supplementary Table S3), AKI was closely related to in-hospital mortality (CA-AKI: *OR* 5.073, 95% CI 2.447–10.517; HA-AKI: *OR* 13.198, 95% CI 8.133-21.419) (Table 2).

## Discussion

In this study, we conducted an epidemiological survey of AKI in a large geriatric population in South China. Age was a risk factor for AKI; in older adults, the overall incidence was 27.8%, which was significantly higher than the incidence in the general population [13,17]. The top three wards with the highest incidence of AKI were the nephrology, respiratory medicine and gastroenterology wards. In addition to traditional risk factors, dementia and tumors were also independently associated with AKI. CA-AKI and HA-AKI patients had longer hospital stays and higher in-hospital costs than patients in the non-AKI group. These two types of AKI also increased the risk of intensive care unit admission, and in-hospital death.

Both AKI and dementia are common in older patients. However, few studies have focused on the relationship between them. Baek et al. found that frailty was associated with an increased risk for AKI in geriatric inpatients, and dementia was an indicator of frailty [19]. It has been suggested that dementia is a risk factor for AKI in the elderly population. Dementia is relatively more common in older adults and has a high incidence [20]. Dementia may lack obvious physical symptoms, and developing countries do not pay enough attention to this silent culprit due to poor health management conditions [21]. A 12-year follow-up cohort study showed that AKI patients were 1.9 times more likely to develop dementia than control subjects [22]. Another study showed that AKI patients who underwent temporary dialysis had twice the risk of developing dementia compared with control subjects [23]. These results suggest that dementia may be an adverse outcome of AKI. Our present study advances the existing results, showing that dementia is also an independent risk factor for AKI. Patients with dementia had a 60.4% increased risk of developing CA-AKI and a nearly 1-fold increased risk of HA-AKI. The relationship between dementia and AKI may be explained by the fact that patients with dementia are likely to have dehydration, drug misuse, and treatment delays [24,25]. These phenomena are more obvious in the out-of-hospital community environment, and we should pay more attention to these patients.

Tumor is another common disease in older patients, and it is also associated with AKI [26]. The mechanisms underlying the development of AKI in patients with tumors include volume factors, infections, the use of iodine contrast agents, the use of chemotherapy drugs and urinary obstruction [26]. In a large population-based study including 1.2 million patients with cancer, the detected incidence rates of AKI in patients with kidney cancer, multiple myeloma, liver cancer, and leukemia were 44%, 33%, 32% and 28%, respectively [27]. Our results supported this previous finding.

AKI significantly increases the clinical burden. These adverse clinical outcomes may be related to the following clinical features of older adults: more tests and monitoring are needed in older adult AKI patients, especially those requiring renal replacement therapy, resulting in increased in-hospital costs; the use of drugs, such as aminoglycosides, renin-angiotensin inhibitors, and iodine contrast agents, are limited in AKI patients, leading to difficulties in the diagnosis and treatment of other comorbidities; and AKI interacts with other diseases of the heart, liver, and central nervous and hematopoietic systems [28], making the condition more serious and complicated.

The incidence of AKI in the Epidemiology of AKI in Chinese Hospitalized adults (EACH) study was 10.9%. After adjusting the SCr test frequency, the incidence was 15.4% in the 65–80-year-old group and 22.2% in the older than 80-year-old group [8]. The incidence of AKI was 27.8% in our study, which was markedly higher than that in the EACH study. The diagnosis of AKI requires measurement of serum creatinine levels at least twice, and the low frequency of creatinine detection can cause misdiagnosis and underestimation of the incidence of AKI. The higher incidence of AKI in our study might be due to frequent creatinine measurements. In this study, 88% of patients had serum creatinine levels measured twice or more, which is much more frequent than the repeated detection rate of 25.3% in China and 63.2–67.6% in developed countries [29]. Therefore, our detection rate is closer to the actual incidence of AKI in older adults in the real world.

There are several limitations. First, urine output data were not used in diagnosing AKI, which would lead to a certain number of missed diagnoses. Second, this study was a retrospective study, lacking information regarding dehydration, infections, potentially nephrotoxic drugs and so on, so we could not correct for these confounding factors that may lead to AKI. Moreover, we were unable to observe the long-term prognosis of the subjects.

## Conclusions

In summary, more than one-fourth of the patients in the geriatric medical wards had AKI. In addition to traditional risk factors, dementia and tumors may be relatively common risk factors for AKI in older adults. AKI was associated with hospitalization time, length of hospital stay, critical care and in-hospital mortality in older adults. The above results provide evidence for the integrated management of AKI in older adults. Furthermore, the long-term outcome of AKI and measures to prevent AKI in older adults should be the focus of future research.

## Supplementary Material

Supplemental Material

Supplemental Material

Supplemental Material

## Data Availability

The datasets analyzed during the current study are available within the article and supplementary file.

## Abbreviations

AKI: acute kidney injury
CKD: chronic kidney disease
ACE: acute care for the elderly
ICD: International Classification of Disease
