# Supplementary material for: Clinical features, risk factors, and clinical burden of acute kidney injury in older adults
Source: Ren Fail. 2020 Nov 16;42(1):1127–34. doi: 10.1080/0886022X.2020.1843491 (PMC7671701; doi:10.1080/0886022X.2020.1843491)
Supplement: Supplemental Material [file IRNF_A_1843491_SM1900.pdf]

Table S1. Clinical characteristics of the study subjects with P>0.05

	Non-AKI (n=4,422)	CA-AKI (n=389)	HA-AKI (n=1,315)	P-value
Myocardial infarction	242(5.5)	19(4.9)	84(6.4)	0.362
Peripheral vascular disease	2,486(56.2)	228(58.6)	767(58.3)	0.305
Cerebrovascular disease	2,504(56.6)	228(58.6)	769(58.5)	0.410
Ulcer disease	188(4.3)	17(4.4)	69(5.2)	0.307
Mild liver disease	2,692(60.9)	244(62.7)	779(59.2)	0.388
Diabetes mellitus without chronic complications	1,550(35.1)	136(35.0)	463(35.2)	0.993
Hemiplegia	8(0.2)	0	3(0.2)	0.646
Leukemia	14(0.3)	1(0.3)	5(0.4)	0.910

AKI, acute kidney injury; CA, community-acquired; HA, hospital-acquired.
