# Supplementary material for: Clinical features, risk factors, and clinical burden of acute kidney injury in older adults
Source: Ren Fail. 2020 Nov 16;42(1):1127–34. doi: 10.1080/0886022X.2020.1843491 (PMC7671701; doi:10.1080/0886022X.2020.1843491)
Supplement: Supplemental Material [file IRNF_A_1843491_SM1882.pdf]

Table S2. Clinical characteristics in different wards

	Gastroenterology (N=1,289)	Respiratory Medicine (N=1,824)	Neurology (N=709)	Nephrology (N=720)	Endocrinology (N=583)	Cardiology (N=1,001)
**Age** [median (Q25, Q75)]	84 (79-87)	84 (80-87)	84 (79-87)	86 (82-89)	83 (75-86)	80 (70-86)
**Charlson comorbidity index score** [median (Q25, Q75)]	4 (2-6)	3 (2-5)	4 (3-5)	5 (3-6)	4 (2-5)	3 (1-5)
**Comorbidities**						
Hypertension (%)	818 (63.5)	1133 (62.1)	534 (75.3)	525 (72.9)	432 (74.1)	731 (73.0)
Myocardial infarction (%)	31 (2.4)	78 (4.3)	26 (3.7)	21 (2.9)	32 (5.5)	157 (15.7)
Congestive heart failure (%)	112 (8.7)	260 (14.3)	94 (13.3)	176 (24.4)	64 (11.0)	485 (48.5)
Peripheral vascular disease (%)	737 (57.2)	802 (44.0)	618 (87.2)	463 (64.3)	349 (59.9)	512 (51.1)
Cerebrovascular disease (%)	738 (57.3)	806 (44.2)	618 (87.2)	469 (65.1)	351 (60.2)	519 (51.8)
Dementia (%)	137 (10.6)	224 (12.3)	150 (21.2)	73 (10.1)	38 (6.5)	30 (3.0)
Chronic pulmonary disease (%)	310 (24.0)	1044 (57.2)	131 (18.5)	201 (27.9)	149 (25.6)	227 (22.7)
Connective tissue disease (%)	128 (9.9)	104 (5.7)	12 (1.7)	29 (4.0)	20 (3.4)	10 (1.0)
Ulcer disease (%)	129 (10.0)	78 (4.3)	12 (1.7)	22 (3.1)	16 (2.7)	17 (1.7)
Mild liver disease (%)	786 (61.0)	989 (54.2)	555 (78.3)	435 (60.4)	470 (80.6)	480 (48.0)
Diabetes mellitus without chronic complications (%)	367 (28.5)	463 (25.4)	343 (48.4)	244 (33.9)	399 (68.4)	333 (33.3)
Hemiplegia (%)	3 (0.2)	3 (0.2)	3 (0.4)	1 (0.1)	1 (0.2)	0
Moderate/severe renal disease (%)	89 (6.9)	105 (5.8)	82 (11.6)	297 (41.3)	97 (16.6)	86 (8.6)
Diabetes mellitus with chronic complications (%)	26 (2.0)	21 (1.2)	19 (2.7)	46 (6.4)	139 (23.8)	20 (2.0)
Non-metastatic tumor (%)	446 (34.6)	432 (23.7)	55 (7.8)	237 (32.9)	71 (12.2)	52 (5.2)
Leukemia (%)	5 (0.4)	5 (0.3)	6 (0.8)	4 (0.6)	0	0
Lymphoma (%)	18 (1.4)	23 (1.3)	4 (0.6)	24 (3.3)	5 (0.9)	2 (0.2)
Moderate/severe liver disease (%)	76 (5.9)	23 (1.3)	1 (0.1)	18 (2.5)	4 (0.7)	1 (0.1)

Metastatic solid tumor (%)	118 (9.2)	173 (9.5)	11 (1.6)	47 (6.5)	20 (3.4)	2 (0.2)
