# Supplementary material for: Clinical features, risk factors, and clinical burden of acute kidney injury in older adults
Source: Ren Fail. 2020 Nov 16;42(1):1127–34. doi: 10.1080/0886022X.2020.1843491 (PMC7671701; doi:10.1080/0886022X.2020.1843491)
Supplement: Supplemental Material [file IRNF_A_1843491_SM1879.pdf]

Table S3. Relationship between AKI and adverse outcomes according to one-way logistic regression

	Need for intensive care		P-value	In-hospital mortality		P-value
	OR	95% *CI*		*OR*	95% *CI*	
**Female**	0.848	0.544-1.322	0.468	0.873	0.599-1.274	0.482
**Age (years)**						
60 - 74	Reference			Reference		
75 - 89	0.706	0.453-1.099	0.123	2.430	1.367-4.319	0.002
≥ 90	0.706	0.382-1.303	0.265	2.478	1.276-4.814	0.007
**AKI**						
Non-AKI	Reference			Reference		
CA-AKI	10.986	6.787-17.782	<0.001	8.510	10.677-32.088	<0.001
HA-AKI	4.879	3.186-7.472	<0.001	30.686	20.427-46.098	<0.001
**Events**						
Required dialysis	88.423	40.590-184.007	<0.001	80.830	37.182-175.717	<0.001
Required intensive care	*			30.686	20.427-46.098	<0.001
**Comorbidities**						

Hypertension	0.668	0.464-0.982	0.030	1.164	0.829-1.634	0.382
Myocardial infarction	3.005	1.800-5.017	<0.001	1.186	0.637-2.207	0.591
Congestive heart failure	1.299	0.852-1.981	0.224	1.272	0.884-1.831	1.195
Peripheral vascular disease	0.453	0.313-0.655	<0.001	0.744	0.547-1.013	0.060
Cerebrovascular disease	0.447	0.309-0.646	<0.001	0.753	0.553-1.024	0.070
Dementia	0.429	0.188-0,979	0.044	0.702	0.396-1.243	0.225
Chronic pulmonary disease	0.789	0.531-1.173	0.242	0.966	0.696-1.340	0.835
Connective tissue disease	1.547	0.777-3.081	0.215	1.793	1.024-3.138	0.041
Ulcer disease	1.107	0.483-2.538	0.810	0.931`	0.433-2.005	0.856
Mild liver disease	0.622	0.434-0.890	0.009	0.966	0.706-1.322	0.828
Diabetes mellitus without chronic complications	0.681	0.455-1.019	0.062	0.832	0.597-1.159	0.276
Hemiplegia	#			#		
Moderate/severe renal disease	1.148	0.896-1.471	0.274	1.440	1.195-1.736	<\0.001
Diabetes mellitus with chronic complications	0.853	0.517-1.410	0.536	1.304	0.966-1.761	0.083

Non-metastatic tumor	1.334	1.100-1.618	0.003	2.020	1.730-2.358	<0.001
Leukemia	#			1.371	0.500-3.759	0.539
Lymphoma	#			1.214	0.678-2.173	0.515
Moderate/severe liver disease	1.297	0.979-1.745	0.104	1.748	1.448-2.109	<0.001
Metastatic solid tumor	1.157	1.058-1.265	0.030	1.488	1.407-1.573	<0.001

AKI, acute kidney injury; CA, community-acquired; HA, hospital-acquired; OR, odds ratio; 95% CI, confidence interval.

*This variable was not included in the logistic regression analysis of the outcomes.

#There were few positive cases, and logistic regression analysis was not performed.
